# Exposure to *Euphorbia lathyris *latex resulting in alkaline chemical injury: a case report

**DOI:** 10.1186/1752-1947-3-115

**Published:** 2009-11-10

**Authors:** Alexander S Ioannidis, Konstantinos I Papageorgiou, Petros S Andreou

**Affiliations:** 1 Department of Ophthalmology, Mid Essex NHS Trust, Court Road, Chelmsford, Essex CM1 7ET, UK

## Abstract

**Introduction:**

We report the case of a patient with extreme pain following accidental exposure to the latex of *Euphorbia lathyris*.

**Case presentation:**

A 76-year-old Caucasian woman attended the ophthalmology department with acute severe bilateral eye pain. This occurred immediately after having pulled a weed out of her garden with her bare hands. She recalled having subsequently rubbed her eyes. The offending plant, was brought into hospital and was identified as the Caper Spurge (*Euphorbia lathyris*). Her ocular pH was alkaline (pH 9). After copious irrigation, the pH normalised. She was treated with topical steroids, cycloplegics, lubricants and opioid oral analgesia. Three days later, she was symptom-free and her vision had returned to normal.

**Conclusion:**

Exposure to Caper spurge latex is a rare cause of keratoconjunctivitis. It can, however, potentially lead to corneal ulceration, anterior uveitis and rarely blindness. Treatment remains largely empirical. Exposure to the milky latex can result in extreme pain requiring prompt treatment. The use of goggles and gloves is recommended when handling this plant.

## Introduction

*Euphorbia lathyris *(Caper spurge) is a common biennial garden plant. It is prevalent in southern England but can occur throughout Europe, North America and Australia. It is known by other names such as the Mole Plant, Gopher Spurge and Myrtle Spurge.

## Case presentation

A 76-year-old Caucasian woman attended the ophthalmology department with acute severe bilateral eye pain. She gave a history of having pulled a large weed from her garden with her bare hands and subsequently rubbing her eyes.

**Figure 1:**
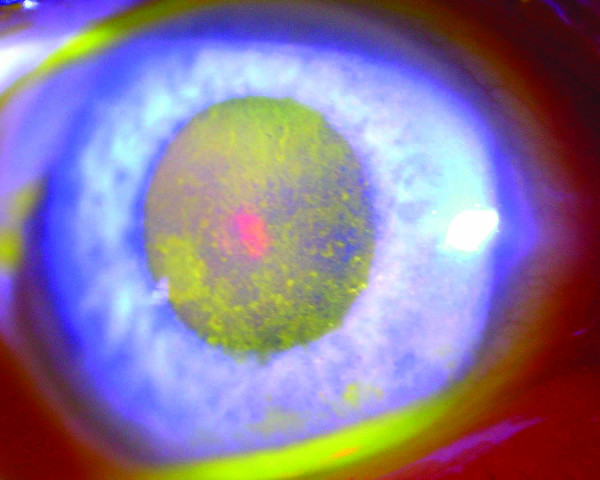
**Photograph of the right eye indicating extensive toxic epitheliopathy following contact with *Euphorbia lathyris *latex**.

On examination, she was in severe distress, complaining of excruciating ocular pain, while pacing in the corridor of the clinic. She had intense bilateral blepharospasm and had been unable to irrigate her eyes before arrival at the hospital. Her visual acuity was 20/125 OD and 20/80 OS but we were unable to perform pinhole examination. On slit lamp examination, she had bilateral conjunctival injection and multiple punctuate erosions on the corneas (Figure 1). There was no stromal thickening or epithelial sloughing. The anterior chambers were quiet. Intraocular pressures were 16 mmHg bilaterally. The ocular pH was checked and found to be alkaline (pH 9) in both eyes. She received immediate irrigation with normal saline 0.9% and the pH normalised (pH 7.0) after 8 litres were instilled in both eyes.

She noted a degree of relief and was treated with hourly dexamethasone 0.1%, cyclopentolate 1% tid, celluvisc 2 hourly and oral vitamin C 1000 mg once daily. Despite a normalised pH, she continued to feel severe pain and required admission to hospital. She received regular oral opioid analgesia overnight.

**Figure 2:**
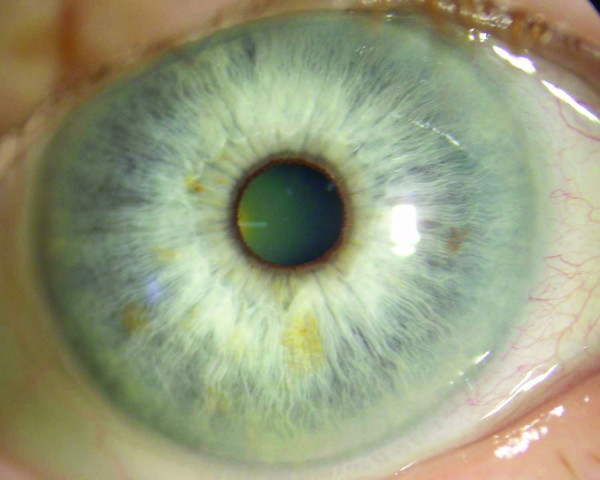
**Photograph of the right eye indicating complete resolution of toxicity 3 days after contact with the irritant latex**.

**Figure 3:**
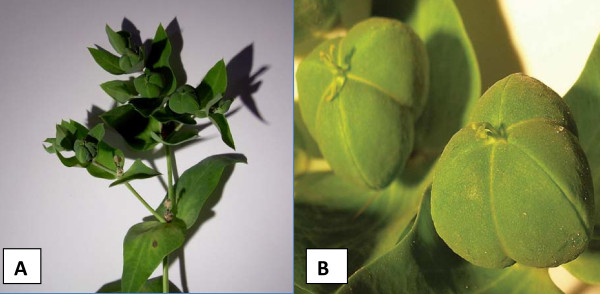
**(A) Photograph of the Caper Spurge (*Euphorbia lathyris*)**. (B) Detail of the characteristic fruiting body resembling a small caper.

Her condition was reviewed the following morning and she was pain-free. On examination 3 days later, her vision was 20/20 in both eyes. The corneas were clear with no areas of epithelial sloughing (Figure 2). The anterior chambers were quiet. The offending plant was presented to the hospital and was subsequently identified as *Euphorbia lathyris *also known as the Caper Spurge (Figure 3a, b).

## Discussion

The Euphorbiaceae includes over 1500 species of trees, succulents, and herbaceous plants [1]. The milky latex of many *Euphorbia *plants is toxic, and may cause severe inflammation of the skin and the eye [2,3]. Ocular inflammation can range from mild conjunctivitis, to severe keratouveitis, and blindness [2,4]. There are other reports in the literature of corneal injury following contact with plant species known to produce irritant saps. In one instance, a patient developed a crystalline keratopathy that resolved spontaneously after 3 months. This resolution was confirmed on confocal microscopy [5].

In cases of suspected corneal contact with the sap of *Euphorbia *sp., there are published recommendations for treatment. These include immediate irrigation, followed by a full ocular assessment. Treatment should include the use of topical antibiotics and cycloplegics. Follow-up should be frequent in the first few days to identify secondary sequelae early such as bacterial supra-infection and uveitis. It is recommended that, where possible, patients should provide a sample of the offending plant for identification purposes.

As far as we are aware, this is the first reported case where the ocular pH has been found to be alkaline following accidental contact with the sap of *E. lathyris*. Although the pH was alkaline initially, we also believe that her extreme distress was caused by another unidentified factor in the milky sap of the plant.

## Conclusion

This report indicates that there is a risk of considerable ocular injury following contact with the latex of *E. lathyris*. In cases of latex exposure, we therefore recommend the regular assessment of ocular pH before and after irrigation to ensure the complete elimination of the milky sap from the ocular surface. It is also recommended that some form of eye protection and gloves should be used when handling these plants to minimize the risk of accidental injury.

## Competing interests

The authors declare that they have no competing interests.

## Authors' contributions

AI was involved in the management of the patient and initiated the preparation of the manuscript. KP performed the literature search and was a contributor in writing the manuscript. PA was also a contributor involved in editing the manuscript. All authors read and approved the final manuscript.

## Consent

Written informed consent was obtained from the patient for publication of this case report and any accompanying images. A copy of the written consent is available for review by the Editor-in-Chief of this journal.
